# The Time Course of Contextual Effects on Visual Word Recognition

**DOI:** 10.3389/fpsyg.2012.00285

**Published:** 2012-08-20

**Authors:** Chia-Ying Lee, Yo-Ning Liu, Jie-Li Tsai

**Affiliations:** ^1^Brain and Language Laboratory, The Institute of LinguisticsAcademia Sinica, Taipei, Taiwan; ^2^Laboratory for Cognitive Neuropsychology, National Yang-Ming UniversityTaipei, Taiwan; ^3^Institute of Cognitive Neuroscience, National Central UniversityJhongli, Taiwan; ^4^Department of Psychology, National Chengchi UniversityTaipei, Taiwan

**Keywords:** anterior N1, contextual effect, event-related potentials, lexical access, P200

## Abstract

Sentence comprehension depends on continuous prediction of upcoming words. However, when and how contextual information affects the bottom-up streams of visual word recognition is unknown. This study examined the effects of word frequency and contextual predictability (cloze probability of a target word embedded in the sentence) on N1, P200, and N400 components, which are related to various cognitive operations in early visual processing, perceptual decoding, and semantic processing. The data exhibited a significant interaction between predictability and frequency at the anterior N1 component. The predictability effect, in which the low predictability words elicited a more negative N1 than high predictability words, was only observed when reading a high frequency word. A significant predictability effect occurred during the P200 time window, in which the low predictability words elicited a less positive P200 than high predictability words. There is also a significant predictability effect on the N400 component; low predictability words elicited a greater N400 than high predictability words, although this effect did not interact with frequency. The temporal dynamics of the manner in which contextual information affects the visual word recognition is discussed. These findings support the interactive account, suggesting that contextual information facilitates visual-feature and orthographic processing in the early stage of visual word processing and semantic integration in the later stage.

## Introduction

Studies have used various measures to demonstrate how the processing of a word can be influenced by its preceding context. In behavioral studies, readers are usually faster or more accurate in responding to words that are congruent with its preceding context (Stanovich and West, 1981; Duffy et al., 1989). By recording eye movements during natural reading, the fixation, and gaze durations are usually shorter for highly expected words than for unexpected words that are embedded in the sentences (Kliegl et al., 2004; Rayner et al., 2004; Sereno et al., 2006; Dambacher et al., 2008; Hand et al., 2010). These results suggest that sentence comprehension depends on continuous prediction of upcoming words. However, *when* and *how* contextual information affects bottom-up streams of visual word recognition is unknown. The temporal resolution of the event-related potentials (ERPs) technique has been used to detail the time course of language comprehension by using a series of ERP components to index various stages of lexical processing (Van Petten and Kutas, 1990; Dambacher et al., 2006; Sereno et al., 2006; Federmeier, 2007; Hand et al., 2010; Molinaro et al., 2010). This study attempted to delineate the manner in which contextual information modulates word recognition during sentence comprehension, especially in the early stage of word processing.

Word recognition models usually assume that a mental lexicon is associated with a “pool” of mentally stored information. Lexical access describes retrieval of such information or access to a discrete lexical entry, either through a search procedure (Forster and Chambers, 1973) or by activating a threshold based on features extracted from the stimulus (Morton, 1969). In general, visual word recognition can be subdivided into three stages, as follows: *prelexical*, *lexical*, and *post-lexical processing* (Forster, 1981; Fodor, 1983). There have been different perspectives on whether contextual information affects word recognition at an early stage, at the moment of lexical access, or at the post-lexical stage of lexical processing. The ***modular view*** proposes that processing at one level of representation must be completed before the output of this processing can be combined with information from other processing levels (Forster, 1981; Fodor, 1983). The word processing in the sentences must be bottom-up driven (i.e., initiated only after the physical properties of the stimuli are received). The context can only exert its effect at post-lexical stage of the word processing for semantic integration. The facilitation effect of the word in the context is simply because it is easier to integrate upon receipt. Consequently, the modular view does not predict interactions between frequency and predictability, especially in the early stage of lexical processing.

An alternative view is the ***interactive account*** (Morton, 1969; McClelland and Rumelhart, 1981), which lacks informational encapsulation, and predicts the immediate and mutual influence at various levels of lexical processing. The contextual information can be used in an anticipatory or predictive manner, and exert its effect from the early stage of word recognition, such as the early perceptual features analysis, to the later stage of lexical activation and selection (Federmeier, 2007). This account allows the features of upcoming words to be pre-activated during online sentence processing as a result of top-down contextual processing. Thus, both frequency and context may affect early stages in word recognition. The facilitation effect of a word in the context may be attributed to the contextual information, which can be used to activate words prior to receiving them. Considering the fundamental difference between the modular and interactive accounts regarding the timing of the influence of information from one linguistic level of representation on the processing of another level, the ERPs technique is particularly suitable in evaluating these distinct claims between modular (integrative) and interactive (predictive) views of language comprehension.

In the ERP literature, the effect of context was usually evaluated by manipulating the degree of fit or semantic congruency between the context and its upcoming word (Kutas and Hillyard, 1980a,b, 1984), predictability (Van Petten and Kutas, 1990; Dambacher et al., 2006; Dambacher and Kliegl, 2007), or sentential constraint (Hoeks et al., 2004; Meyer and Federmeier, 2007) in various studies. Despite the various ways to term the contextual influences, empirically, these are usually determined by the cloze procedure, in which participants were asked to complete a sentence fragment with a word that first comes to their mind. The cloze probability of a word refers to the percentage of people who completed a sentence frame with that particular word (Taylor, 1953). A well-replicated finding in the ERP literatures is that N400 amplitudes are inversely proportional to the cloze probability. For example, in the following sentence from the study by Kutas and Hillyard (1984): “*He liked lemon and sugar in his tea/coffee*,” the higher the cloze probability of a word (*tea*) in a context, the more reduced the amplitude of the N400 compared to an unexpected word (*coffee*). In general, the reduction of N400 amplitudes is found with words that can be easily integrated into the preceding word, sentence, or discourse context (Kutas and Hillyard, 1980a,b; Van Petten and Kutas, 1990, 1991; van Berkum et al., 1999). These types of findings suggest that the N400 component is sensitive to the processing of lexical integration. The facilitation of processing words in a sentence reflects the ease of integrating the word into context, or the extent to which the context pre-activates specific properties of those words.

To further examine the manner in which contextual information modulates word level processing, studies manipulated the contextual constraint, and the lexical properties of an upcoming word, such as word frequency. Among all types of lexical properties, the word frequency effect has been recognized for its robust influence on the process of word recognition. Relative to high frequency words, readers tend to require a longer period of time to respond to low frequency words in naming and lexical decision tasks (Forster and Chambers, 1973), and require longer fixation and gaze time on low frequency words in natural sentence reading (Inhoff and Rayner, 1986; Kliegl et al., 2004). Some studies manipulating word frequency and word predictability propose the possible mechanism of contextual influence on parafoveal preview. For instance, Hand et al. (2010) showed the word frequency and word predictability interaction on duration measures when considering the launch distance. The word predictability effect was stronger for low frequency words than for high frequency words at the near launch site, but the effect was stronger for high frequency words than for low frequency words. Tracking the word frequency effect across behavioral and electrophysiological paradigms is particularly relevant because its presence is considered a marker for successful lexical access (Embick et al., 2001; Sereno and Rayner, 2003; Hand et al., 2010). Since the word frequency effect has been used to determine the point in time of lexical access, the earliest word frequency effect on ERPs provides an upper limit for the latency of lexical access.

The ERP data has clearly demonstrated that, when all other factors are constant, N400 amplitude is an inverse function of the eliciting frequency of a word (Bentin et al., 1985; Rugg, 1990). In addition, the N400 frequency effect interacts with a variety of other factors that influence the ease of semantic processing, such as repetition, word position in the sentences, and the predictability of the word in the sentences. For example, the repeated presentation of a word in the word list can reduce or eliminate the N400 frequency effect (Rugg, 1990). Van Petten and Kutas (1990) revealed that the effect of frequency on N400, in which low frequency words elicited larger N400 than high frequency words, was found only when the word occurred early in the sentence, but not at the end of the sentence. Given that the word position may reflect the build-up of context “online,” the interaction between word frequency and word position may imply that the frequency effect of lexical processes can be superimposed by the contextual constraint of the sentence. However, it is important to note that a later-occurring word position in a sentence does not necessarily imply that there is increased contextual constraint. Dambacher et al. (2006) further examined the effects of frequency, predictability, and position of words during word-by-word sentence reading. Congruent with Van Petten and Kutas (1990), this study found interactions of predictability and frequency, as well as of position and frequency on N400. The N400 amplitude exhibited a larger predictability effect for low frequency than for high frequency words, and suggested that semantic contextual constraints can override N400 frequency effects (Embick et al., 2001; Dambacher et al., 2006). In addition, a strong frequency effect was observed on the frontocentral P200, in which the P200 amplitude was smaller for high frequency words than for low frequency words. By treating frequency as an index for lexical access, the authors claimed that lexical access was presumably completed for high frequency words within the first 200 ms after stimulus presentation during sentence reading, whereas low frequency words were being processed. This also explained the larger predictability effect on the N400 for low frequency words than for high frequency words. This occurred because the lexical access of low frequency words benefits from contextual information during the N400 time window, and this benefit is strongly reduced in the processing of high frequency words that were previously recognized.

Recent studies also claimed that early processing makes contact with lexical entries for words that include semantic and phonological properties; therefore, lexical frequency, semantic features, and lexicality affect neural computation within 200 ms post-stimulus onset in a visual word recognition task (Sereno et al., 1998, 2003; Hauk et al., 2006a,b; Penolazzi et al., 2007; Scott et al., 2009). Sereno et al. (2003) examined the temporal locus of contextual influence on word frequency and word ambiguity, and revealed that the contextual effect, coincident with frequency effect, was found on the N1 component from 132 to 192 ms post-stimulus. The ERP literature usually considers the N1 as an index of the visual signal associated with the early stage of word recognition. The findings of Sereno et al. (2003) suggest that the context affects the selection of the appropriate meaning of an ambiguous word in the early stage of lexical processing, which supports the interactive view. Penolazzi et al. (2007) demonstrated the word frequency and probability effects at 120 and 180 ms after written word onset. Other studies also found frequency by predictability interaction (Dambacher et al., 2006; Hauk et al., 2006b; Penolazzi et al., 2007) and semantic coherence effect (Hauk et al., 2006a) as early as approximately 130 ms in the early stage of lexical processing. Federmeier and Kutas (2001) also demonstrated that context begins to have its effects very early in frontal N1, which peaked at around 150 ms, and that this influence continues into the early and late N400 time windows. The effect of constraint on the response to expected exemplars begins in the N1 time window, with a reduced N1 to expected exemplars in high- as opposed to low-constraint sentences. These results indicate that semantic context integration may occur at an early stage, and almost simultaneously with the processing of information regarding the form and lexical properties of a word.

Most studies claim that the early semantic effects on lexical access are mainly based on early effects of lexicality and word frequency (Sereno et al., 2003; Dambacher et al., 2006; Hauk et al., 2006b; Penolazzi et al., 2007; Scott et al., 2009). However, both lexicality and word frequency are highly correlated with the word-form properties (such as bigram, trigram, and word-form frequencies). These early effects maybe attributed to word-form recognition, rather than actual lexical access. Penolazzi et al. (2007) orthogonally manipulated the length, the lexical frequency, and the cloze probability of a word that occurred in a specified semantic context, and found that frequency and probability effects were modulated by word length at 120 and 180 ms after written word onset. Particularly, the long and short words exhibited opposite word frequency effects in the early time window, which may explain the lack of early word-related ERP effects in earlier studies if the physical properties, such as word length, were not controlled effectively. Penolazzi et al. (2007) found that the word length interacts with both frequency and cloze probability during the early time window, but not on the N400 component. The main effect of cloze probability was found on the N400 and post-N400 time windows, and these late ERP indexes are insensitive to stimulus variance. Although the contextual influence starts at the early time window, these early neuropsychological markers depend mainly on the perceptual or other prelexical features of the stimuli. Thus, the contextual effect on early ERP components, such as N1 or P200, may not imply that access to lexico-semantic information occurs within the first stages of lexical access, but acts in an anticipatory or predictive manner for the early perceptual features analysis.

This is further supported by Solomyak and Marantz (2009), who examined the visual recognition of heteronyms to distinguish the abstract word-form process from actual lexical access in the brain. Heteronyms (e.g., “wind,” which has two distinct meanings depending on the pronunciation) are phonological and semantically distinct words that share a common orthography, which provides a unique opportunity to distinguish between the processing of lexical property (the frequency ration of one meaning to the other) and word-form properties (open bigram, trigram, and whole-word-form frequencies) in the early stages of processing. Their data revealed a considerable effect of the form properties of the heteronym in the left hemisphere on the M170 and of heteronym frequency ration on the M350. The true lexical properties of heteronyms did not affect processing until after 300 ms post-stimulus, which supports the late access theory. This finding also suggests that the early frequency effect, as reported in previous literature, may only reflect abstract word-form identification rather than actual lexical access (Solomyak and Marantz, 2009).

Related literature has consistently demonstrated that the N400 amplitude is sensitive to the expectancy of a word in a semantic context. However, it remains unclear whether the effects of contextual influence or lexico-semantic processing can be found in early components, such as N1 or P200. A few studies have demonstrated these early ERP effects under the influence of physical or prelexical variables, such as word length, bigram, and trigram frequencies (Hauk and Pulvermüller, 2004; Hauk et al., 2006a). In alphabetic writing systems, word frequency is usually confounded with word length. Most studies that manipulate word frequency have carefully controlled the word length. However, in some cases, the mixed usage of words with various lengths or other physical factors is unavoidable (such as using a corpus of sentences as the stimulus set in Dambacher’s series of studies) which may affect or attenuate the short-lived early ERP effects, since these early components are typically focal and brief. Thus, physical properties of the stimulus must be efficiently controlled or explicitly considered to clarify the functional characteristics of these early ERP effects in sentence comprehension.

The English and Chinese writing systems differ in their orthographic features, and the manner in which these features map onto the phonological structure of words. English is an alphabetical language that uses letters and letter combinations to represent the sounds of words. By contrast, the Chinese writing system uses square-shaped characters as the basic reading unit that links directly to monosyllabic sounds, but not to phonemes. Most importantly, according to the Chinese word corpus of Academia Sinica Balanced Corpus (2004), over 76% of the words (type) consist of two or three characters. This study used the advantages of Chinese two-character compounds, which allowed us to bypass the natural confound between word frequency and word length in alphabetic writing systems, to delineate the nature of predictive processing mechanisms in sentence comprehension, especially in the early stage of lexical processing. The effects of contextual predictability (cloze probability) and word frequency of the two-character words in the middle of the sentence are measured during the time windows of the anterior N1, P200, and N400. The target words will be presented in two sessions in order to counterbalance their appearances at high and low predictable contexts. To reduce the effect from repetition, participants were required to come back for the second session at 2 weeks later. The repetition effects will also be examined to evaluate if the repeated presentation of targets would cause any effect on these ERPs components. This allows researchers to determine the functional stage of word recognition in which the contextual information begins to interact with bottom-up processing of visually presented sentence completions.

## Materials and Methods

### Participants

Twenty-one right-handed native Chinese speakers (eight males) were paid to participate in this experiment (mean age = 23.6 years, range: 18–29 years), and had no history of neurological or psychiatric disorders. All participants were native Chinese speakers with normal or corrected-to-normal vision. Written consent was obtained from all participants.

### Experimental design and materials

The contextual predictability (high versus low) and word frequency of the target word were manipulated in a two-by-two factorial design (see Table 1). One hundred two-character words were chosen as target words from a Chinese corpus (2004). There were an equal number of high- and low frequency words among the target words, 50 words for each condition. The mean word frequency per million was 91.25 (Max: 294.07; Min: 13.84) for high frequency target words, and 1.59 (Max: 7.27; Min: 0.23) for low frequency target words. The words in high- and low frequency conditions were further matched for the visual complexity and orthographic neighborhood size. Two types of sentences were constructed for each target word. Therefore, 200 sentences containing 25 or 26 characters were generated, and a target word was embedded at the 11th to 16th character positions of each sentence. When constructing the sentences, care was taken to avoid the lexical associate word of the target appearing in the context preceding the target. The cloze probability of the target was assessed by 19 participants who did not participate in the ERP experiment. In the cloze task, the participants were presented with sentence fragments preceding the target words, and were asked to fill in a word that first came to their mind to complete each sentence fragment. The predictability value was calculated based on the proportion of raters (19 participants) who filled in the target words as their first answers. Each list consisted of 100 sentences, in which half of the target words were highly predictable, and the other half were less predictable. For the high predictability condition, the mean cloze probability value was 0.80 for high frequency predictable target words, and 0.75 for low frequency predictable target words. For the low predictability condition, the mean cloze probability value was 0.05 for high frequency predictable target words, and 0.07 for low frequency predictable target words. For each participant, two lists were created to counterbalance the predictability and frequency of the target word in each sentence. For each type of predictability, half of the targets were high frequency words, and half were low frequency words. The target words in high- and low frequency conditions were matched for the number of strokes (*t* = 1.31, *p* = 0.17). For the word preceding the target word, the statistical analyses revealed that there were no significant difference in their word frequency (raw and log frequency, *F*s < 1) and word classes (*X*2 = 2.994, *p* = 0.81) among four conditions. Each participant read the two lists in two separate experimental sessions. To prevent the repetition effect on the target words, participants were required to return to the second session at least 2 weeks after the first session.

**Table 1:** **Means of word frequency and predictability of target words and example sentences (Chinese, word-by-word translation, and whole sentence translation) for each condition**.

Condition		Word frequency	Log frequency	Predictability	Example sentences
HF	HP	91.25	2.84	0.80	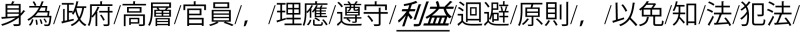
					As/government/senior/officials/ 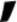 /should/obey/***interest***/avoid/principle/ 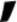 /lest/know/laws/violate the laws/
					As senior officials in government, one should avoid conflict of ***interests*** lest we consciously violate the laws.
	LP	91.25	2.84	0.01	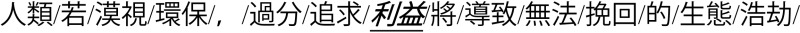	
					Human/if/ignore/environment/ 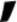 /excessively/pursue after/***interest***/will/cause/can’t/reverse/DE/ecological/catastrophe/
					If human keep ignoring the environmental issues and pursuing of ***interests*** excessively will, it would lead to irreversible ecological catastrophe.
LF	HP	1.59	0	0.75	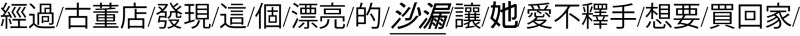	
					Come over/antique shop/find/this/GE/beautiful/DE/***sandglass***/make/her/be foud of it/want to/buy it home/
					She passed by an antique shoe and was fond of the beautiful ***sandglass***, which make her wants to buy it home.
	LP	1.59	0	0.01	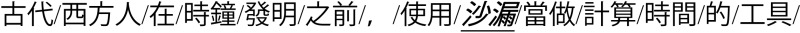
					Ancient time/Western/at/clock/invent/before/ 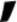 /use/***sandglass***/as/measure/time/DE/tool/
					Before the clock was invented, the ancient Western uses ***sandglasses*** for timing.

*Target words are highlighted with bolds and underlines in the example sentences. HF, high frequency; LF, low frequency; HP, high predictability; LP, low predictability*.

### Procedure

Participants were individually seated at a distance of approximately 70 cm in front of a monitor, in an electrically shielded room. Each participant received 12 trials for practice and 100 randomized experimental trials in four test blocks. Participants were allowed to rest between test blocks for as long as they required. For each trial, a fixation cross was presented in the center of the screen for 500 ms as a warning that a sentence was about to begin. Sentences were subsequently presented one word at a time at the center of the screen. The size of each character presented on the screen was 32 × 32 pixels, and there was a space of 4 pixels between characters. The width of a character and the space before it subtended 0.9° of visual angle. Each word appeared for 250 ms, and was followed by a blank screen for 450 ms. Participants were asked to read for comprehension, and tried not to blink during this period of time. A total of 29% of sentences were followed by a comprehension question. Participants were asked to answer by clicking the left or right button on the mouse for Yes and No responses. Otherwise, participants started the next trial by pressing the left mouse button. Across participants, an average of 98.3% of comprehension questions were answered correctly (Max: 100%; Min: 87.3%).The entire session lasted for approximately 40 min.

### EEG recording and preprocessing

The electroencephalogram (EEG) was recorded from 64 sintered Ag/AgCl electrodes (QuickCap, Neuromedical Supplies, Sterling, TX, USA) with a common vertex reference located between Cz and CPz. The EEG was continuously recorded and digitized at a rate of 500 Hz. The signal was amplified by SYNAMPS2 (Neuroscan, Inc., El Paso, Texas, USA) with a low-pass filter of 100 Hz for off-line analysis. The data were re-referenced off-line to the average of the right and left mastoids for further analysis. Vertical eye movements were recorded by a pair of electrodes placed on the supraorbital and infraorbital ridges of the left eye, and horizontal eye movements were recorded by electrodes placed lateral to the outer canthus of the right and left eyes. A ground electrode was placed on the forehead anterior to the FZ electrode. Electrode impedance remained below 5 kΩ.

For off-line analysis, the continuous EEG was epoched with 100 ms before the onset of the target word, and 700 ms post-stimulus intervals. The pre-stimulus interval (−100 to 0 ms) was used for baseline correction. Trials contaminated by eye movement or with voltage variations larger than 60 μV were rejected. The band-pass filter of 0.1 and 30 Hz (zero phase shift mode, 12 dB) was used. The ERPs were calculated for each participant and each condition for every electrode.

## Result

**Figure 1:**
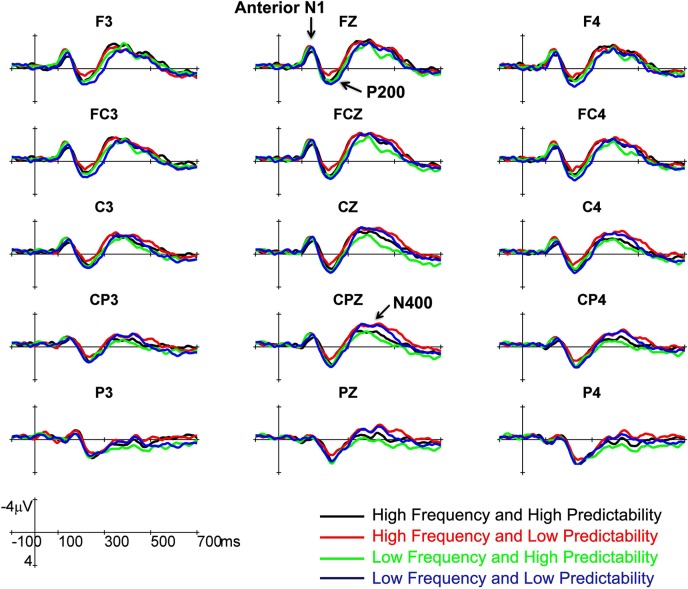
**The grand averaged ERPs to the high- and low frequency target words in high- and low predictability contexts across two sessions from 15 representative electrodes**. Major ERPs components for further analysis are labeled.

Figure 1 shows the grand averaged ERPs to the high- and low frequency target words in high- and low predictability contexts across two sessions from representative electrodes. Visual inspection of the data revealed three main components in all conditions for further analysis. The first distinct negative peak was the anterior N1, which peaked at approximately 100 ms at frontocentral sites. It was followed by the P200, which was a positive-going wave that reached its peak at approximately 220 ms, and was most prominent at the frontocentral electrodes. The third component was the N400, a negative deflection following the N1-P200 complex, which peaked at approximately 350 ms with central-parietal distribution. Effects of word frequency and predictability were accessed by comparisons of mean amplitudes in the following three time windows of interest: anterior N1 (120–150 ms), P200 (200–250 ms), and N400 (300–500 ms).

The repeated measures of ANOVA were performed on these ERPs components, with factors of predictability (high versus low), word frequency (high versus low), repetition (session 1 versus session 2), and electrodes in the region of interest. The examination of repetition effect was used to determine if the repeated presentation of target word in two sessions would cause any repetition effect, and in particular, if any interaction would be caused by the repetition. Thus, the data of two sessions would be merged for each ERP component, if the repetition effect did not interact with condition effects. Otherwise, only the data of the first session would be analyzed if the repetition effect interacted with condition effects. For each ANOVA, the Greenhouse–Geisser adjustment to the degrees of freedom was applied to correct violations of sphericity associated with repeated measures. Consequently, the corrected *p*-value was reported for all *F* tests with more than one degree of freedom in the numerator. The *post hoc* tests were conducted by using Tukey’s procedure.

### Anterior N1

**Figure 2:**
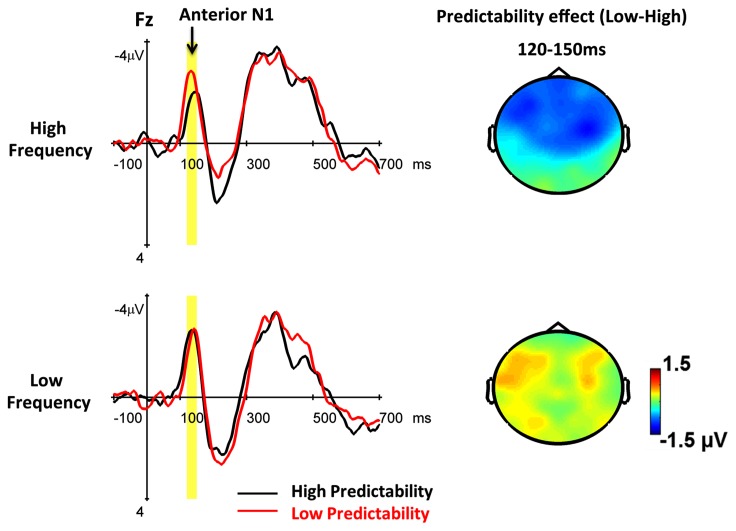
**The grand average ERPs words embedded in high and low predictability contexts under high and low frequency conditions across two sessions are shown at Fz**. The yellow area indicates the time window (120–150 ms) of the anterior N1. The topographic maps show the predictability effects (low minus high) for high and low frequency words, respectively.

The mean amplitude of the N1 was analyzed by a four-way ANOVA with predictability (high and low), word frequency (high versus low), repetition (Session 1 versus Session 2), and electrode (FZ, FCZ, CZ, F3/4, FC3/4, C3/4) as within-subject factors. The mean amplitudes for all conditions are presented in Table 2. The choice of electrodes was motivated by previous studies that reported the contextual effects on the frontal N1 (Federmeier and Kutas, 2001; Dambacher et al., 2006). The main effect of repetition was not significant (*F* < 1). The repetition effect failed to demonstrate any significant interaction with predictability or with word frequency (*F*s < 1). The data from Session 1 and Session 2 were merged for further analysis by a three-way ANOVA with predictability (high and low), frequency (high and low), and electrode (FZ, FCZ, CZ, F3/4, FC3/4, C3/4) as within-subject factors. The data revealed that the main effects of both predictability and word frequency were insignificant (*F*s < 1). The two-way interaction between predictability and word frequency [*F*(1, 20) = 5.45, *p* = 0.030] was significant, whereas three-way interaction among predictability, word frequency, and electrode was insignificant [*F*(8, 160) = 1.63, *p* = 0.17]. The *post hoc* test revealed that low predictability words elicited a larger negativity than high predictability words in the reading of high frequency words [*F*(1, 20) = 8.77, *p* = 0.007], but not in the reading of low frequency words [*F*(1, 20) = 2.33, *p* = 0.14] (see Figure 2).

**Table 2:** **Mean amplitudes of anterior N1 for each condition and each electrode of interest in each session**.

N1 (120–150 ms)		Session 1						Session 2					
		High frequency			Low frequency			High frequency			Low frequency		
		Left	Midline	Right	Left	Midline	Right	Left	Midline	Right	Left	Midline	Right
High predictability	F	−1.07	−1.90	−1.50	−2.62	−2.96	−2.37	−1.22	−1.68	−1.35	−1.54	−2.03	−1.80
	FC	−1.15	−1.60	−1.21	−2.58	−2.92	−2.12	−1.58	−1.91	−1.55	−1.73	−2.02	−1.87
	C	−0.80	−0.93	−0.44	−2.23	−2.29	−1.76	−1.29	−1.45	−1.02	−1.36	−1.35	−1.51
Low predictability	F	−1.99	−2.50	−2.35	−1.80	−2.70	−1.92	−2.33	−2.77	−2.15	−1.46	−1.96	−1.38
	FC	−1.86	−2.31	−2.19	−1.95	−2.67	−1.84	−2.61	−2.80	−2.42	−1.53	−1.99	−1.27
	C	−1.43	−1.58	−1.72	−1.79	−2.17	−1.51	−2.24	−2.32	−2.05	−1.07	−1.27	−0.73

### P200

**Figure 3:**
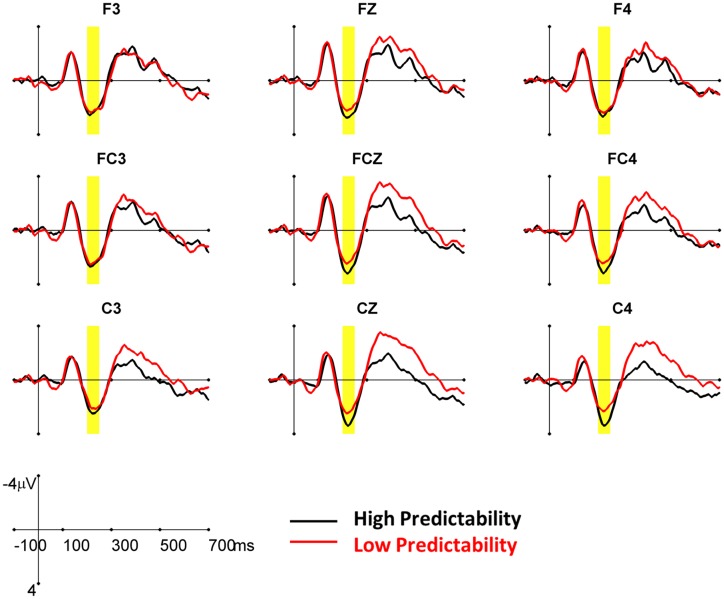
**The grand average ERPs for target words embedded in high and low predictability conditions from the first sessions are shown at nine frontocentral electrodes**. The yellow area indicates the time window (200–250 ms) of the P200 component.

Previous studies have suggested a frontocentral distributed contextual effect on the P200 (Federmeier et al., 2005; Dambacher et al., 2006). Accordingly, nine left anterior electrodes (FZ, FCZ, CZ, F3/4, FC3/4, C3/4) were chosen for analyzing the P200. The mean amplitude of the P200 was analyzed by a four-way ANOVA with predictability (high and low), word frequency (high versus low), repetition (Session 1 versus Session 2), and electrode (FZ, FCZ, CZ, F3/4, FC3/4, C3/4) as within-subject factors. The data revealed a significant three-way interaction among repetition, predictability, and frequency [*F*(1, 20) = 4.73, *p* < 0.05]. Therefore, only the data from Session 1 was further analyzed by a three-way ANOVA with predictability (high and low), frequency (high and low), and electrode (FZ, FCZ, CZ, F3/4, FC3/4, C3/4) as within-subject factors. The mean amplitudes for all conditions are presented in Table 3. The data failed to demonstrate significant main effects of predictability [*F*(1, 20) = 1.89, *p* = 0.18] and word frequency [*F*(1, 20) = 2.32, *p* = 0.14]. The only significant interaction was the two-way interaction between predictability and electrode [*F*(8, 160) = 2.74, *p* = 0.03]. The *post hoc* analysis revealed that the high predictability words elicited more positive P200 than low predictability words did in most of the frontocentral electrode (*p*s < 0.001), except for F3[*F*(1, 160) = 0.55, *p* = 0.458], FC3 [*F*(1, 160) = 1.21, *p* = 0.274], and F4 [*F*(1, 160) = 1.72, *p* = 0.192] (see Figure 3).

**Table 3:** **Mean amplitudes of P200 for each condition and each electrode of interest in each session**.

P200 (200–250 ms)		Session 1						Session 2					
		High frequency			Low frequency			High frequency			Low frequency		
		Left	Midline	Right	Left	Midline	Right	Left	Midline	Right	Left	Midline	Right
High predictability	F	2.32	2.36	2.23	2.32	2.83	2.63	1.49	1.57	1.51	1.70	1.37	1.38
	FC	2.37	2.67	2.56	2.66	3.20	3.23	1.28	1.66	1.61	1.42	1.42	1.23
	C	2.14	2.70	2.97	2.48	3.42	3.15	1.31	1.70	1.82	1.37	1.78	1.28
Low predictability	F	1.92	1.94	1.86	2.51	2.20	2.64	0.29	0.10	0.83	2.58	2.73	2.84
	FC	1.99	1.94	1.80	2.71	2.56	2.82	0.38	0.58	0.94	2.38	2.80	2.92
	C	1.77	1.94	1.72	2.17	2.59	2.55	0.57	1.11	1.41	2.24	2.98	3.00

### N400

Based on previous ERP studies that have examined the semantic processing of Chinese words (Lee et al., 2007; Hsu et al., 2009; Huang et al., 2011), the analysis of mean N400 amplitude was conducted separately for data derived from the midline and lateral sites using the four-way ANOVAs with predictability (high and low), word frequency (high versus low), repetition (Session 1 versus Session 2), and electrode in the region of interest as within-subject factors. Five electrodes (FZ, FCZ, CZ, CPZ, and PZ) were selected for midline N400 analysis. For N400s in lateral electrode sites, 10 electrodes (F3/4, FC3/4, C3/4, CP3/4, and P3/4) were chosen as the electrode variable. The mean amplitudes for all conditions are presented in Table 4. The midline analysis revealed a significant main effect of repetition [*F*(1, 20) = 5.29, *p* < 0.05]. The repetition by predictability interaction [*F*(1, 20) = 6.58, *p* < 0.05] and the repetition by frequency interaction [*F*(1, 20) = 5.11, *p* < 0.05] were also significant. A similar pattern was also found in the lateral analysis. Therefore, only the data from the first session was used for further analysis with the three-way ANOVA with predictability (high and low), frequency (high and low), and electrode in the region of interest as within-subject factors.

**Table 4:** **Mean amplitudes of N400 for each condition and each electrode of interest in each session**.

N400 (300–500 ms)		Session 1						Session 2					
		High frequency			Low frequency			High frequency			Low frequency		
		Left	Midline	Right	Left	Midline	Right	Left	Midline	Right	Left	Midline	Right
High predictability	F	−1.61	−2.00	−1.62	−1.61	−1.37	−0.93	−3.25	−3.90	−3.00	−2.23	−2.66	−2.03
	FC	−1.31	−1.71	−1.46	−1.07	−1.18	−0.61	−3.19	−3.93	−2.88	−2.18	−2.47	−2.02
	C	−0.78	−1.39	−0.84	−0.64	−0.79	−0.32	−2.42	−3.21	−1.86	−1.49	−1.75	−1.42
	CP	0.33	−0.18	0.14	0.27	−0.29	0.48	−1.07	−2.64	−0.94	−0.17	−1.46	−0.24
	P	1.68	0.72	1.37	2.01	0.85	1.78	0.54	−0.60	0.68	1.45	0.65	1.77
Low predictability	F	−1.65	−2.30	−1.98	−1.51	−2.76	−1.72	−2.35	−3.57	−2.35	−1.48	−2.13	−1.44
	FC	−1.83	−2.57	−2.07	−1.55	−2.92	−1.85	−2.65	−3.73	−2.72	−1.84	−2.49	−1.93
	C	−1.66	−2.51	−2.14	−1.81	−2.96	−2.16	−2.56	−3.40	−2.48	−1.46	−2.36	−1.56
	CP	−0.78	−1.72	−0.75	−0.97	−2.17	−1.13	−1.21	−3.13	−1.64	−0.55	−2.12	−0.89
	P	0.85	−0.68	0.26	1.09	−0.89	0.35	0.29	−1.13	0.23	0.58	−0.39	0.72

**Figure 4:**
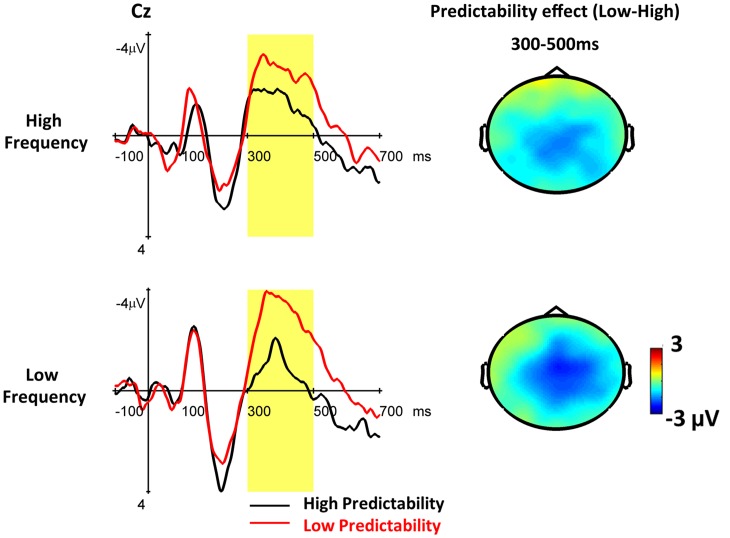
**The grand average ERPs to words embedded in high and low predictability contexts under high and low frequency conditions from the first sessions are shown at Cz**. The yellow area indicates the time window (300–500 ms) of the N400. The topographic maps show the predictability effects (low minus high) for high and low frequency words, respectively.

The midline analysis revealed a significant predictability effect [*F*(1, 20) = 15.82, *p* = 0007] (see Figure 4). Low predictability words elicited more negative N400 responses than high predictability words. However, the frequency effect was not significant (*F* < 1). A significant predictability by electrode interaction was observed [*F*(1, 20) = 5.33, *p* = 0.009]. The predictability effects were significant at FCZ, CZ, CPZ, and PZ (*p*s < 0.0001), but only marginally significant at Fz (*p* = 0.06). The two-way interaction between frequency and predictability [*F*(1, 20) = 1.03, *p* = 0.32] and the three-way interaction among frequency, predictability, and electrode [*F*(4, 80) = 1.02, *p* = 0.37] were not significant. Planned comparisons revealed that the predictability effect was significant at low frequency words [*F*(1, 20) = 11.42, *p* = 0.003], but it was only marginally significant at high frequency words [*F*(1, 20) = 3.78, *p* = 0.066]. For the lateral analysis, both the main effects of frequency and predictability (*F*s < 1) and their interaction [*F*(1, 20) = 1.04, *p* = 0.32] were not significant. All other interactions also failed to reach significance (*F*s < 1).

## Discussion

This study investigated *when* and *how* context influences word recognition, especially in the early stage of lexical processing. The cloze probability and the word frequency effects of the two-character Chinese compound embedded in the middle of the sentences were measured in relation to a set of ERP components (anterior N1, P200, and N400) to index various stages of lexical processing. The data revealed predictability effects on the anterior N1, P200, and N400 components, but demonstrated a differing modulation effect to word frequency and long-term repetition (the same set of target words were embedded in various sentence frames with a 2-week interval). Repetition did not modulate the predictability effect on the anterior N1, whereas it significantly reduced and changed the predictability effects on the P200 and N400. In the literature, the anterior N1 usually indexes the early stage of perceptual analysis, whereas the N400 reflects the retrieval of the lexical item from semantic memory. The differing sensitivity to long-term repetition is consistent with previous studies, which demonstrated that the perceptual properties were less affected by the long-term repetition, whereas the representational changes involved in semantic decisions about previously encountered stimuli may last for several days within the semantic network (Meister et al., 2007). Moreover, the interactions between predictability and word frequency were only found on the anterior N1, but not on the P200 and N400. The different patterns on the anterior N1, P200, and N400 suggest that contextual effects may differ at various stages of lexical processing.

The data demonstrated a significant predictability-by-frequency interaction on the anterior N1 and suggests that contextual information exerts its effect within 100–200 ms after perceiving the upcoming word. Unlike the typical finding on the N400, the predictability effect, in which the low predictability words elicited a more negative N1 than high predictability words, was only found in reading high frequency words, but not in reading low frequency words. Our findings are in general congruent with the early frequency or contextual effects (within 200 ms) that have been reported in a number of studies (Federmeier and Kutas, 2001; Sereno et al., 2003; Dambacher et al., 2006, 2009; Dikker et al., 2009; Kim and Lai, 2011) and suggest a top-down influence on pre-activated form-based representations. Studies have used the RMS analysis to identify at least two brain responses for the word-evoked potential that occurs within 200 ms after perceiving a word, peaking at 100–120 ms and at 160–180 ms (Hauk et al., 2006a; Penolazzi et al., 2007). The grand average scalp topographies of these peak activations usually exhibit a frontocentral negativity, the anterior N1, and a posterior positivity, the posterior P1, for the first peak, and approximately the opposite polarity pattern for the second peak, the N170. These early contextual or semantic ERP effects that occur within 200 ms are usually short-lived and topographically specific, and thus are much more vulnerable than the widely distributed long-lasting late ERP effects. Therefore, there are some inconsistencies among these early ERP effects in their time windows and spatial distribution, including the anterior N1, the posterior P1, and the N170. Several studies have suggested that the early top-down modulation might originate from the visual cortex. For instance, Sereno et al. (2003) reported that sentence context modulated the ERP in the posterior regions elicited by ambiguous words 132–192 ms after stimulus onset. Dikker et al. (2009) reported the effect of syntactic expectedness on visual M100 at occipital cortex, in which the unexpected item elicited an enhanced M100 relative to the expected controls, but only when word category was overtly marked by a functional morpheme, supporting the hypothesis that the early visual responses to word-forms can be influenced by prior syntactic context. Kim and Lai (2011) examined the time course of interactions between lexical semantic and word-form analysis during reading of sentences, in which the target word might be replaced by pseudowords which either did or did not orthographically resemble a contextually supported real word, or could be replaced by non-word consonant strings. The pseudowords resembling the contextually supported real words elicited an enhanced occipital distributed P130 relative to real words. The pseudowords that did not resemble the contextually supported real words elicited an enhanced N170 relative to non-word consonant strings. These findings support the view of a top-down excitation of form features in the information flow within visual cortex.

Other studies, however, suggested that the early contextual effects may occur in regions other than the visual cortex. For example, Federmeier and Kutas (2001) reported a contextual effect on the anterior N1 for picture processing, in which the expected example showed a reduced anterior N1 relative to unexpected examples, but only for high constraint sentences. Hauk et al. (2006a) examined the cortical activation elicited by words and pseudowords that varied in orthographic typicality (the frequency of their component letter pairs (bigrams) and triples (trigrams). The typicality effect was found within 100 ms after stimulus onset, in which words and pseudowords with atypical orthography elicited a stronger activity in left peri-sylvian areas (regions extending from Wernicke’s area to the posterior/inferior parietal cortex and prefrontal cortex) than those with typical orthographic patterns. However, the lexicality (words versus pseudowords) did not interact significantly with the orthographic typicality until 160 ms. The findings suggest a series of distinct but interactive processing stages in word recognition, from the early form-based analysis to the later lexico-semantic processes. This is further supported by the study of Dambacher et al. (2009), which demonstrated an early predictability effect that was found at approximately 100 ms at right anterior and left posterior sites. In sum, these findings support a top-down influence on early feature processing, in which the context may afford form-specific predictions for the upcoming stimuli.

The other possibility for the early contextual effect might be that the context plays a role in directing attention to specific sensory features early in the information processing stream. In the literature, the attentional effect over the N1 commonly shows a posterior distribution (central, parietal, and occipital), but an anterior distributed N1 (central and frontal) has also been reported (Luck and Hillyard, 1995; Luck et al., 2000; Vogel and Luck, 2000; Tollner et al., 2009). In the visual domain, two types of N1 responses can be found: an early anterior N1 (which occurred over frontocentral electrodes and peaked approximately 120 ms post-stimulus) and a somewhat later posterior N1 (which occurred over lateral posterior electrodes and peaked approximately 175 ms post-stimulus for contralateral stimuli; Mangun and Hillyard, 1991; Mangun et al., 1993; Luck and Hillyard, 1995). In general, both the anterior N1 and the posterior N1 reflect a benefit of correctly allocated attentional resources, and are manifestations of a crucial sensory attention-gating mechanism. For example, in the visual cueing paradigm, the N1 amplitude is largest for perceptual features in attended (versus unattended) locations and on attended (versus unattended) objects. It suggests that perceptual features are only selected for further perceptual processing if they are in attended locations or on attended objects (Anllo-Vento and Hillyard, 1996; Martinez et al., 2006). In addition, the effect of modality change was most pronounced on the anterior N1 and almost disappeared on the central-posterior N1. This suggests that the anterior N1 enhancement may reflect the detection of a modality change and the initiation of the attentional readjustment, in order to optimize target detection (Tollner et al., 2009).

Lee et al. (submitted) examined the contextual predictability (cloze probability of the final word in the sentence) and orthographic similarity (identical words, orthographically similar homophones, and orthographically dissimilar homophones) of the final words, in an online sentence comprehension task. The data revealed an interaction between predictability and orthographic similarity on the anterior N1. Orthographic similarity only had an effect on the anterior N1 with high predictability sentences, in which an identical character elicited a greater N1 than both orthographically similar and dissimilar homophones. In other words, a larger N1 was evident when the expected character was presented. However, this is only true in reading high predictability sentences. This can be further supported by the current findings, in which the predictability effect can be obtained when the upcoming words are of high frequency. Based on the logogen model (Morton, 1969), a large number of passive word-detector elements (logogens) can accrue information or be activated from a number of sources in parallel. Word frequency and contextual information both may act to reduce the amount of stimulus information that was required to exceed the threshold frequency of a logogen, by lowering the threshold and by raising the level of activation. Assuming a high frequency words would maintain a relatively high resting state in the system, it is easier for the high frequency word to reach the threshold and to except its effect (such as becoming available to capture attention or to achieve lexical access) with the help of context. Our findings are compatible with the early selection model of attention, which contends that attention acts as a sensory gain mechanism that enhances perception of the expected stimuli.

The current data revealed a significant predictability effect on the P200, in which the low predictability words elicited a less positive P200 than high predictability words. Recent studies have demonstrated that the P200 is larger (more positive) for strongly constrained sentence endings, regardless of whether the actual word was the expected word, especially for right, but not left, visual field presentations (Federmeier et al., 2005; Wlotko and Federmeier, 2007). These findings suggest that the contextual effect occurred in the early time window. Dambacher et al. (2006) demonstrated a significant predictability effect on the P200. However, contradictory to our findings and those of other studies, they found a more positive P200 for low predictability words in the sentences. It is important to note that, in their data, the word position also strongly modulates the P200, which was not included as factor in this initial analysis. In fact, there was a high correlation between word predictability and word position (*r* = 0.41) in the sentence corpus that they used. When effects of predictability and position were estimated within one model, neither the word predictability nor the predictability-by-frequency interaction affected the P200 amplitudes. The only significant factor influencing the P200 was the word position, in which the P200 was larger for words at the beginning and end of sentences than for words in the middle of sentences (Dambacher et al., 2006). This was unexpected because our findings and those of other studies have suggested that the P200 varies with the level of expectancy for a particular item in a sentence. According to Dambacher et al. (2006), the increased working memory load or alertness in the middle of the sentence might be possible reasons for the decreasing P200 amplitude toward the center of a sentence and for subsequently increasing amplitudes. However, further studies are needed to examine this explanation. Indeed, in the literature on the visual search paradigm, the P200 has been used to index the mechanisms for selective attention, feature detection (including color, orientation, and shape), and the early stage of item encoding (Luck and Hillyard, 1994). In general, decreased amplitude of the P200 results from increased attention, which decreases the amount of search space and facilitates feature classification in visual search during the perceptual processing. While recording eye movements during natural reading, the fixation, and gaze durations are usually shorter for highly predictable words than for lowly predictable words that are embedded in the sentences (Kliegl et al., 2004; Rayner et al., 2004; Sereno et al., 2006; Dambacher et al., 2008; Hand et al., 2010). These might reflect the amount of attention to be allocated to the words in the sentences. The lowly predictable words require more attention for further processing, thus eliciting less positive P200s than the highly predictable ones. Taken together, the P200 may reflect the matching of input with expectation. The contextual information can be used to predict or to pre-activate the expected word, thereby facilitating the perceptual matching process in the early stage.

In the N400 time window, the interaction between frequency and predictability was not significant. However, the *post hoc* comparison revealed a significant predictability effect for low frequency words and a marginally significant predictability effect (*p* = 0.06) for high frequency words. The overall pattern is consistent with previous studies (Van Petten and Kutas, 1990; Dambacher and Kliegl, 2007) in which low predictability words elicited a larger N400 than high predictability words, whereas the predictability effect was stronger for low frequency words than high frequency words. The N400 reflects the brain activity associated with semantic access, and the N400 reduction is regarded as ease of semantic integration. Our data revealed that both high and low frequency words can benefit from the contextual information. However, this benefit is substantially reduced for high frequency words because these are recognized or processed faster and more efficiently in lexical access.

In summary, this study demonstrates contextual predictability effects on the anterior N1, P200, and N400 components. The findings support the interactive account, and suggest that contextual information facilitates visual-feature and orthographic processing in the early stage of word recognition, and semantic integration in the later stage. Similar conclusions were reached by demonstrating the contextual constraining effect on phonological regularity of English indefinite articles (“an” precedes nouns beginning with vowel sounds, whereas “a” precedes nouns beginning with consonant sounds; DeLong et al., 2005) and lexical status (Laszlo and Federmeier, 2009). However, these effects were mainly found in the classical N400 time window. There have been debates on whether the N400 reflects an automatic lexical access or the post-lexical semantic integration. The contextual modulation of the N400 is difficult to differentiate if readers use context to generate expectancies for upcoming items (prediction view) or if they are forced by the words to devote more or fewer resources in integrating words into sentence representations (integration view). Recently, Molinaro et al. (2010) demonstrated a larger N400 for words with neighbors of higher frequency compared to words without such neighbors only when the critical word was embedded in low-constraining sentences. Most importantly, the cloze probability manipulation affects ERPs about 100 ms before the effect of neighbor frequency manipulation (Molinaro et al., 2010). The context facilitates the word recognition even before the lexical competition among a set of word neighbors begins, which thus supports the predictive view. In addition, this study used the anterior N1 and P200 to index the modulation of attention and perceptual analysis in the bottom-up stream of word processing. The interaction between predictability and frequency on the anterior N1 and the main effect of predictability on the P200 provide strong supports for the hypothesis that contextual information can be used to predict and pre-activate the features of upcoming words.

## Conflict of Interest Statement

The authors declare that the research was conducted in the absence of any commercial or financial relationships that could be construed as a potential conflict of interest.

## Supplementary Material

The Supplementary Material for this article can be found online at http://www.frontiersin.org/Language_Sciences/10.3389/fpsyg.2012.00285/abstract
